# Supplementary material for: Endogenous siRNAs promote proteostasis and longevity in germline-less Caenorhabditis elegans
Source: eLife. 2020 Mar 26;9:e50896. doi: 10.7554/eLife.50896 (PMC7136021; doi:10.7554/eLife.50896)
Supplement: Transparent reporting form [file elife-50896-transrepform.docx]

***eLife’s* transparent reporting form**

We encourage authors to provide detailed information *within their submission* to facilitate the interpretation and replication of experiments. Authors can upload supporting documentation to indicate the use of appropriate reporting guidelines for health-related research (see [EQUATOR Network](http://www.equator-network.org/%20)), life science research (see the [BioSharing Information Resource](https://biosharing.org/)), or the [ARRIVE guidelines](http://www.plosbiology.org/article/info:doi/10.1371/journal.pbio.1000412) for reporting work involving animal research. Where applicable, authors should refer to any relevant reporting standards documents in this form.

If you have any questions, please consult our Journal Policies and/or contact us: [editorial@elifesciences.org](mailto:editorial@elifesciences.org).

**Sample-size estimation**

- You should state whether an appropriate sample size was computed when the study was being designed
- You should state the statistical method of sample size computation and any required assumptions
- If no explicit power analysis was used, you should describe how you decided what sample (replicate) size (number) to use

Please outline where this information can be found within the submission (e.g., sections or figure legends), or explain why this information doesn’t apply to your submission:

You can find the information about the sample size and statistical methods in figure legends. Sample size was not computed, but was used as generally practiced in the field (according to published experiments), and is indicated in the figure legend and/or method section. In general experiments were replicated at least three times (independent biological replicates). In lifespan experiments, at least 100 animals were scored. In experiments requiring more laborious manual scoring of animals we endeavored to provide at least 30 worms per group.

**Replicates**

- You should report how often each experiment was performed
- You should include a definition of biological versus technical replication
- The data obtained should be provided and sufficient information should be provided to indicate the number of independent biological and/or technical replicates
- If you encountered any outliers, you should describe how these were handled
- Criteria for exclusion/inclusion of data should be clearly stated
- High-throughput sequence data should be uploaded before submission, with a private link for reviewers provided (these are available from both GEO and ArrayExpress)

Please outline where this information can be found within the submission (e.g., sections or figure legends), or explain why this information doesn’t apply to your submission:

You can find the information about the number of biological replicates in figure legends. In general experiments were replicated at least three times (independent biological replicates).

In lifespan assays, contaminated plates were excluded by censoring, as were any worms that died of bursting, drying out or internal hatching, as per standard methods.

High-throughput sequencing data and microarray data generated and/or analyzed during this study were deposited under the Gene Expression Omnibus, with accession numbers GSE122457 and with GSE128935.

**Statistical reporting**

- Statistical analysis methods should be described and justified
- Raw data should be presented in figures whenever informative to do so (typically when N per group is less than 10)
- For each experiment, you should identify the statistical tests used, exact values of N, definitions of center, methods of multiple test correction, and dispersion and precision measures (e.g., mean, median, SD, SEM, confidence intervals; and, for the major substantive results, a measure of effect size (e.g., Pearson's r, Cohen's d)
- Report exact p-values wherever possible alongside the summary statistics and 95% confidence intervals. These should be reported for all key questions and not only when the p-value is less than 0.05.

Please outline where this information can be found within the submission (e.g., sections or figure legends), or explain why this information doesn’t apply to your submission:

Information about statistical reporting (including statistical tests used, methods of multiple test correction, exact P values, exact values of N , and dispersion and precision measures), can be found in the figure legends in a dedicated material, in table S8 and/or in the figure legends.

(For large datasets, or papers with a very large number of statistical tests, you may upload a single table file with tests, Ns, etc., with reference to sections in the manuscript.)

**Group allocation**

- Indicate how samples were allocated into experimental groups (in the case of clinical studies, please specify allocation to treatment method); if randomization was used, please also state if restricted randomization was applied
- Indicate if masking was used during group allocation, data collection and/or data analysis

Please outline where this information can be found within the submission (e.g., sections or figure legends), or explain why this information doesn’t apply to your submission:

Experimental groups were defined by genotype.

**Additional data files (“source data”)**

- We encourage you to upload relevant additional data files, such as numerical data that are represented as a graph in a figure, or as a summary table
- Where provided, these should be in the most useful format, and they can be uploaded as “Source data” files linked to a main figure or table
- Include model definition files including the full list of parameters used
- Include code used for data analysis (e.g., R, MatLab)
- Avoid stating that data files are “available upon request”

Please indicate the figures or tables for which source data files have been provided:

Raw and processed high-throughput sequencing data and microarray data generated and/or analyzed during this study were deposited under the Gene Expression Omnibus, with accession numbers GSE122457 and with GSE128935.
